# Photonic Needles for Light Delivery in Deep Tissue-like Media

**DOI:** 10.1038/s41598-017-05746-7

**Published:** 2017-07-17

**Authors:** Romy Fain, Felippe Barbosa, Jaime Cardenas, Michal Lipson

**Affiliations:** 1 Electrical and Computer Engineering, Cornell University, Ithaca, NY 14853 USA; 2 Electrical Engineering, Columbia University, New York, NY 10025 USA

## Abstract

We demonstrate a new platform for minimally invasive, light delivery probes leveraging the maturing field of silicon photonics, enabling massively parallel fabrication of photonic structures. These Photonic Needles probes have sub-10 μm cross-sectional dimensions, lengths greater than 3 mm–surpassing 1000 to 1 aspect ratio, and are released completely into air without a substrate below. We show the Photonic Needles to be mechanically robust when inserted into 2% agarose. The propagation loss of these waveguides is low–on the order of 4 dB/cm.

## Introduction

The use of light for biological imaging and stimulation, has an array of useful applications, but due to combined scattering and absorption effects in tissue^1, 2^, current techniques for optical stimulation and detection in deep tissue require relatively large optical probes and fibers to be implanted, causing significant damage to the tissue. Ground-breaking early light delivery probe schemes, (for example, large chip shank-like designs^3–6^, reminiscent of traditional electrode on chip platforms^7, 8^, fiber-like devices^9–13^, or designs with traits of both^14–18^) are designed to reach deep tissue, but have large cross-sections on the order of 100 μm across or more. Such schemes are destructive to the biological tissue, inducing an immunological response, disrupting the system one would want to measure, and impeding the signal response collected^19–22^.

The most notable methods attempting to address invasiveness issues while achieving deep tissue light delivery are multiphoton stimulation^1, 23–28^, and microfabricated multisite light delivery probes^3–6^. Multiphoton light delivery is minimally invasive, but depths greater than 2 mm are not resolvable without the assistance of more invasive methods. Physical light delivery probes can easily reach multiple data collection sites millimeters deep, but at the expense of very invasive, large cross-sections, on the order of 10’s of sites per 10’s of cubic millimeters per probe. The large size of these probes makes scaling beyond a few hundred sites problematic. Attempts have been made to miniaturize these devices^3, 5, 29–32^, but the tradeoffs remain between accessing deep-tissue, and achieving minimally invasive, high performance light delivery.

We show here a platform for light delivery based on high aspect ratio Photonic Needles – free standing optical waveguides that are long enough to reach deep-tissue, but narrow enough to cause minimal damage. These Photonic Needles have a cross-sectional diameter of only 3 to 10 μm across the entire 3.5 mm length of the probe, displacing orders of magnitude smaller volumes than standard methods. This platform leverages the maturing field of silicon photonics, enabling massively parallel fabrication of photonic structures using CMOS processing.

## Methods

**Figure 1:**
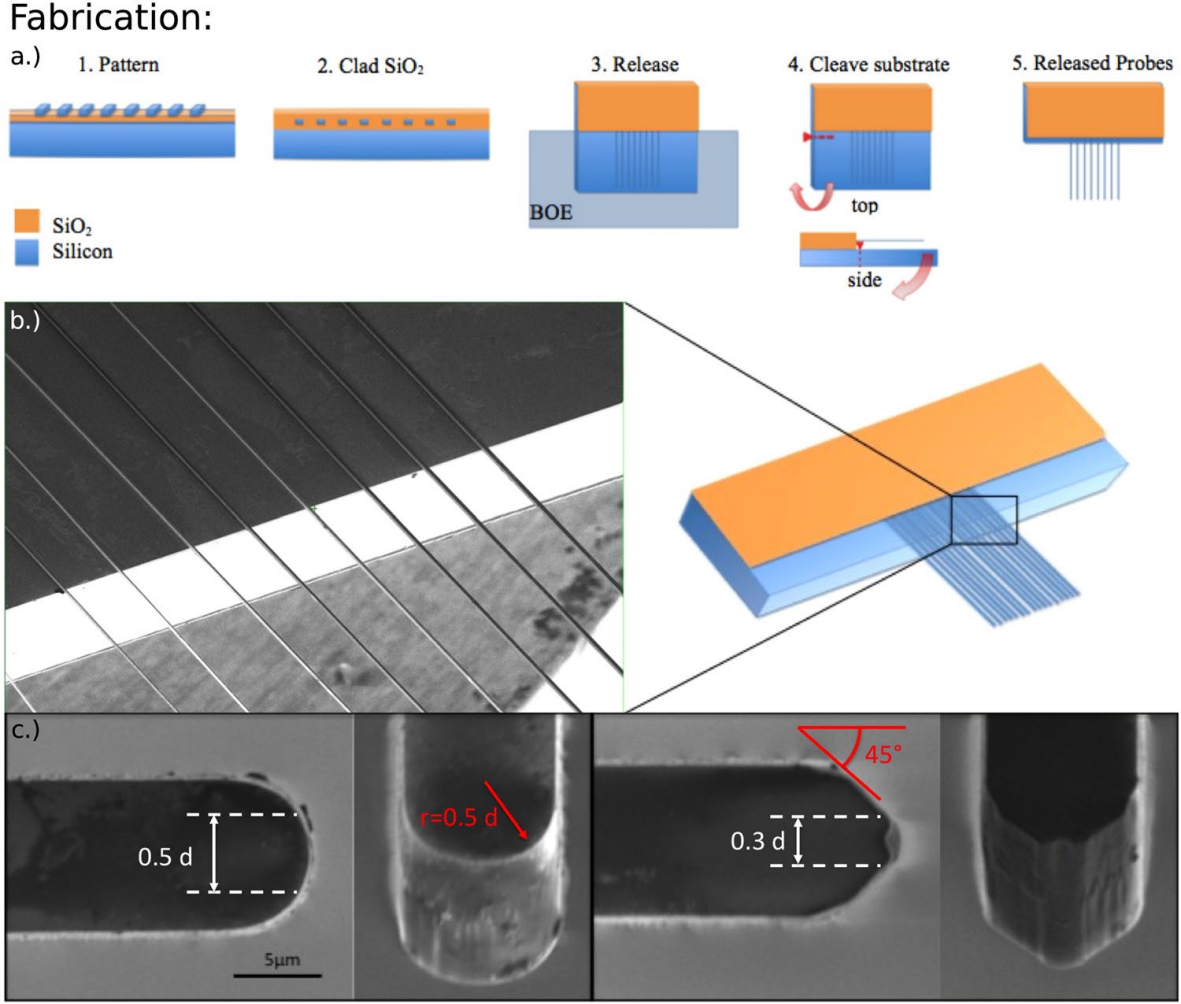
Details of the Photonic Needle fabrication using the Mechanical Substrate Removal (MSR) process. (**a**) Processing steps (left to right): (**1**) patterning of the Photonic Needle waveguides on the SOI wafer, (**2**) cladding the waveguides in PECVD oxide, (**3**) releasing diced chip vertically in BOE, (**4**) scribing at the base of the released section and mechanically cleaving off the substrate, (**5**) finally releasing the probes. (**b**) Scanning Electron Micrograph (SEM) of the Photonic Needles showing the pristine cleaved silicon face, post-MSR, at the interface between the portion of the chip where the needles are released and where they are not released (left). A schematic of the released needles indicating where the SEM was taken is shown on the right. Note that this pristine surface is in contrast to typical rough etched surfaces resulting from long backside etches. (**c**) SEMs of both curved (left) and angled (right) Photonic Needle tips, taken from the top of the chip and at a 45 degree angle relative to the chip. Note that the top faces are much smoother than the sides, showing that the sidewall roughness is primarily due to the initial waveguide etch process and not any part of the release process. The dimensions in white are referring to the approximate effective cross-sections, which mediate the critical force for insertion for each tip type, described in further detail with the data in Fig. 4. The dimensions in red indicate that the patterned radii of all the radiused tips are equal to half the width (d), and that the patterned angle of all the angled tips are at 45 degrees to the length of the needle.

In order to overcome the challenge of fabricating these 1000:1 aspect ratio waveguide probes**, w**e developed a process based on Mechanical Substrate Removal (MSR). The mechanical removal of the substrate (see Fig. 1), overcomes the difficulty of chemically removing a thick substrate that is hundreds of microns thick, while maintaining the integrity of the suspended needle that is only a few microns thick. The extremely long etch times needed to remove the entire substrate can penetrate even thick thermal oxide protective release layers and damage the small waveguides beneath, often etching all the way through especially long, small cross-section devices. It is typically difficult to get high yield fabrication of MEMs cantilevers even with much larger cross-sections and shorter lengths^33–36^. The process we developed involves manually cleaving the substrate away. The process shown in Fig. 1 starts with an SOI wafer of approximately 3.6 μm of thermal oxide buried beneath approximately 10 μm of crystalline silicon. The Photonic Needle waveguides are patterned using contact photolithography in widths ranging from 3 to 10 μm wide. The Photonic Needles are aligned perpendicular to the major flat of the <100> wafer, and therefore perpendicular to the crystallographic plane for cleaving the substrate later in the process. A higher elastic modulus could be achieved by similarly aligning along the crystallographic planes in a <111> wafer. After etching the waveguide needles to the buried oxide, a conformal PECVD oxide cladding is deposited to approximately 3.7 μm thick. Then the wafer is diced into single chips using a silicon dicing blade for lower loss facets. Individual chips are then placed into custom machined Teflon holders, which allow for the chip to be vertically submerged in 6:1 BOE. This releases only the Photonic Needle sections to be inserted into biological media in later experiments, and leaves the oxide cladding intact on the other side of the chip for effective optical coupling into the input facets. This release etch does not damage the Photonic Needles, because the etch rate of silicon in BOE is negligible. Next the chip is critical point dried to avoid stiction in contact with the silicon substrate. At this point, the silicon substrate is carefully mechanically cleaved close to the base of the released waveguide probes via Mechanical Substrate Removal (MSR). This is achieved by marking the edge of the substrate close to the oxide base of the released needles with a diamond scribe, and then pushing down on the substrate and away from the released needles, while holding the base of the chip supported on a stack of microscope slides, a few millimeters above the work surface. This leaves a silicon substrate handle approximately 15 × 5 mm at the base of the Photonic Needles used to insert the entirely released lengths of the needles into the biological tissue. Figure 1b shows the Scanning Electron Micrograph (SEM) of the pristine cleaved silicon face, post-MSR, at the interface between the portion of the chip where the needles are released and where they are not (left). Note that this pristine surface is in contrast to typical rough etched surfaces resulting from long backside etches. Figure 1c shows the SEMs of both curved (left) and angled (right) Photonic Needle tips, taken from the top of the chip and at a 45 degree angle relative to the chip. Note that the top faces are much smoother than the sides, showing that the sidewall roughness is primarily due to the initial waveguide etch process and not any part of the release process. The dimensions in white are referring to the approximate effective cross-sections, which mediate the critical force for insertion for each tip type, described in further detail in the Results and Discussion section below. The dimensions in red indicate that the patterned radii of all the radiused tips are equal to half the width (d), and that the patterned angle of all the angled tips are at 45 degrees to the length of the needle.

## Results and Discussion

**Figure 2:**
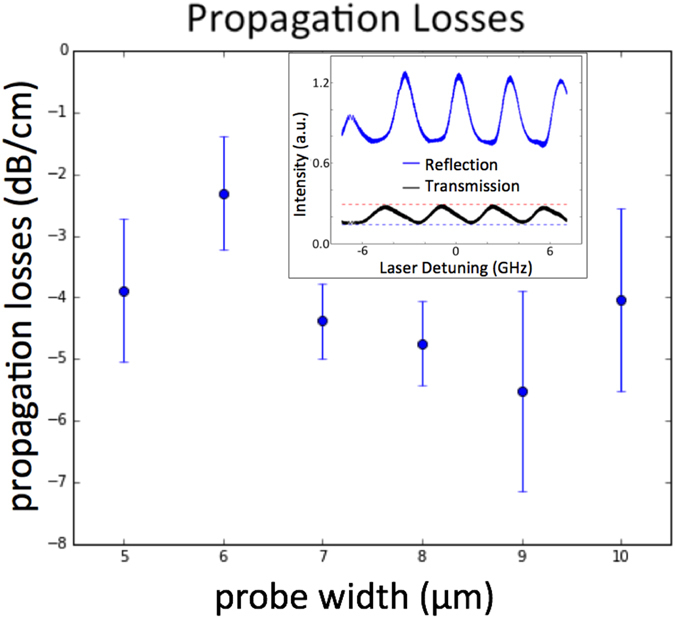
Figure 2 shows the estimated propagation losses for 10 μm thick probes with waveguide widths ranging from 5 to 10 μm wide. The propagation loss was extracted by measuring the transmission and reflectivity of the Fabry-Perot cavity^37–43^ created between blunt polished end facets for a 10.2 μm long probe, where 3.5 μm of the waveguide length is released and 6.7 μm of the waveguide length is clad (inset shows an example of the transmission and the reflection spectra).

The optical quality of the Photonic Needles is high, with measured propagation losses of less than 4(+/−) 2 dB/cm. This loss is primarily due to the sidewall roughness inherent with contact photolithography, and could be easily improved with deep uv or ebeam lithography. Figure 2 shows the estimated propagation losses for 10 μm thick probes with waveguide widths ranging from 5 to 10 μm wide. The propagation loss was extracted by measuring the transmission and reflectivity of the Fabry-Perot cavity created between blunt polished end facets for a 10.2 μm long probe where 3.5 μm of the waveguide length is released and 6.7 μm of the waveguide length is clad (inset shows an example of the transmission and the reflection spectra)^37–43^. The setup consisted of lensed fibers for coupling light in and out of the needles, with a circulator between the source telecom laser at 1550 nm and the needle input facet to measure the reflected intensity data, in addition to the transmitted intensity data collected at the output of the released needle tip. The overall losses were approximately 8 dB on average when coupling with a lensed fiber of 2.5 μm mode field diameter. These losses could easily be improved by minimizing the mode mismatch between the input facets of the Photonic Needle input facets and the fiber input.

We determine experimentally the minimal probe cross-section allowed (such that they can be inserted into biological tissue without mechanically failing) to be on the order of 4 microns. In order to answer the question of how small the probe can be before it buckles, we insert the suspended probes into 0.5% and 2% agarose which have been shown to be similar in mechanical properties to mouse cortex without and with dura intact^44–47^. The chip holding the array of Photonic Needles was fixed to a 3 axis stage adjacent to a second 3 axis stage with a rectangular cube of agarose. The agarose was supported by a structure of microscope slides on all but the side facing the tips of the Photonic Needles. The needles were inserted by manually actuating the micrometer of one stage towards the other.

**Figure 3:**
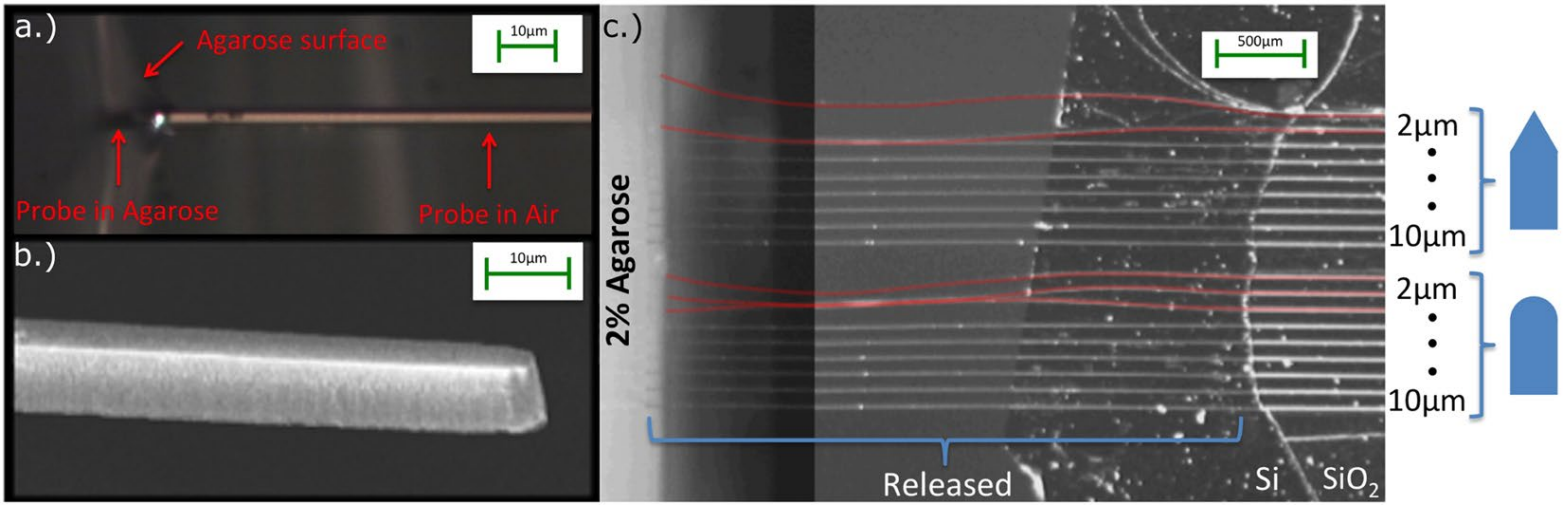
(**a**) Microscope image of a 3.25 mm long suspended probe with 3 × 10 μm cross-section being inserted into 0.5% agarose by weight, to simulate mouse cortex without dura intact^44, 45^. (**b**) SEM image of the probe shown in (**a**) after release and before insertion. (**c**) Composite microscope image of released probes with two different tip geometries and widths varying from 2 to 10 μm being inserted into 2% agarose by weight to simulate mouse cortex with dura intact^44–47^. The Photonic Needles that buckled during insertion are shown in red.

**Figure 4:**
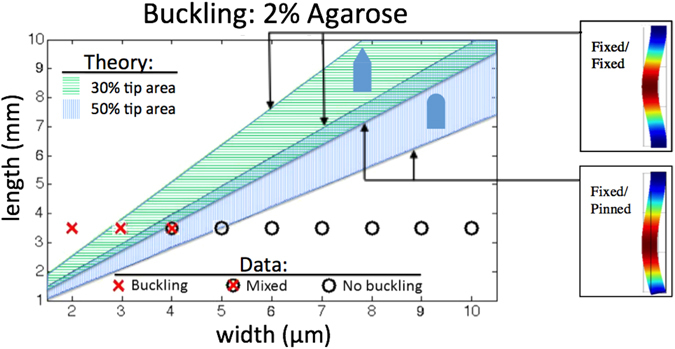
Maximum Photonic Needle lengths above which buckling occurs (for a range of widths). The range of waveguide lengths derived from theory assumes the boundary conditions are somewhere between fixed/fixed and fixed/pinned for the inserted waveguides with both pointed and rounded tips. (,  respectively) Dimensions were extracted from our experiments for buckling and no buckling conditions (,  respectively). The mixed point () represents dimensions where the rounded tip needle () buckled and the pointed tip needle () did not buckle. Right are finite element (FEM) rendered beams buckling for fixed/pinned and fixed/fixed boundary conditions (not to scale). Arrows point to the curves describing each boundary condition assumption enclosing the theoretical range of critical dimensions for each tip geometry.

Figure 3a shows a microscope image of a 3 × 10 μm cross-section probe approximately 3.25 mm long being inserted into 0.5% agarose, with no buckling. Figure 3b is an angled SEM of the same 3 × 10 μm cross-section probe before insertion. Figure 3c is a composite microscope image demonstrating a full double set of released probes being inserted into 2% agarose. One can see that for the angled tip set, the 2 and 3 μm wide needles buckled, but the 4, 5, 6, 7, 8, 9, and 10 μm wide needles did not. For the curved tip set below, the 2, 3, and 4 μm wide needles buckled, but the 5, 6, 7, 8, 9, and 10 μm wide needles did not. Buckling needles are colorized in red. Figure 4 shows the theoretical maximum Photonic Needle lengths (for a range of widths) above which buckling occurs for the inserted waveguides with both angled (green) and rounded (blue) tips and our experimental data. We expect that the tip geometry effects the critical cross sectional dimension, because the angled tip will effectively have a smaller area pushing into the surface of the flexing viscoelastic medium than a rounded tip. The theoretical curves are obtained using Euler’s buckling equation^48–50^

1 $${F}_{critical}=\frac{K{\pi }^{2}EI}{{L}^{2}}$$

where *K* is the column effective length factor determined by the boundary conditions, *E* is the elastic modulus, *I* is the area moment of inertia (=base*height^3^/12), and *L* is the unsupported column length. *F*_*critical*_ is the estimated force needed for insertion in the brain tissue of a mouse cortex with dura intact from previous studies^45, 47^ (scaled for cross-section) to be on the order of 74 μN for a 5 × 10 micron blunt tipped needle. The boundary conditions in our experiments are somewhere in between fixed/fixed (K = 4) and fixed/pinned (K = 2.045) end conditions, since dimpling of the viscoelastic surface will provide some imperfectly rigid resistance to rotation at the tip. Therefore, both boundary conditions are shown bounding the theoretical values expected here. To the right of Fig. 4 are finite element (FEM) rendered beams buckling for fixed/pinned and fixed/fixed boundary conditions (not to scale). The experimental data in Fig. 4 shows the dimensions extracted from our experiments where buckling occurred (red cross) and no buckling occurred (circle). The mixed point represents dimensions where the rounded tip needle buckled and the pointed tip needle did not buckle. From the data, one can see that the widths for which buckling occurred were less than 4(+/−)1 μm, corresponding to what is predicted by the theory.

**Figure 5:**
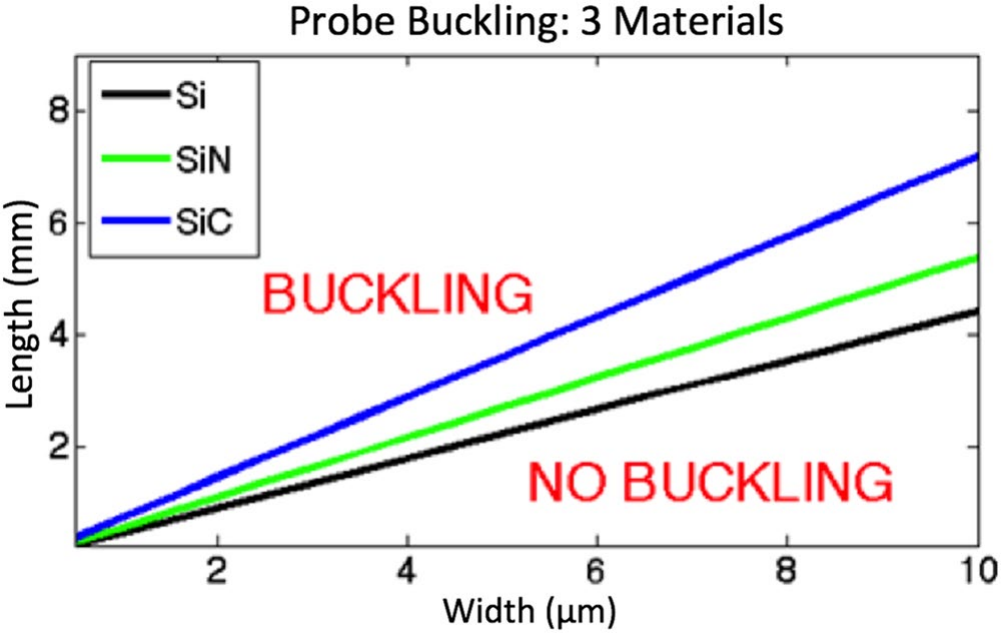
Critical length as a function of cross sectional size, considering silicon (E = 220 GPa), silicon nitride (E = 250 GPa), and silicon carbide (E = 450 GPa). One can see that a 3.5 mm long probe composed of silicon is expected to buckle at a width of approximately 6 μm while the same probe, composed of SiC with the same geometry would not buckle. We assume here a conservative blunt tip geometry, as well as a pinned/fixed boundary condition (K = 2.045) instead of the fixed/fixed boundary condition (K = 4).

We expect that even longer Photonic Needles could be inserted in biological medium without buckling if composed of materials such as silicon nitride and silicon carbide, whose higher elastic moduli (E) contribute to larger stiffnesses. The graph in Fig. 5 shows the critical length as a function of cross sectional size considering silicon (E = 220 GPa), silicon nitride (E = 250 GPa), and silicon carbide (E = 450 GPa). One can see that a 3.5 micron long probe composed of silicon is expected to buckle at a width of approximately 6 microns while the same probe, composed of SiC with the same geometry would not buckle. We assume here a conservative blunt tip geometry, as well as a pinned/fixed boundary condition (K = 2.045) instead of the fixed/fixed boundary condition (K = 4).

## Conclusion

We demonstrate the concurrent mechanical and optical robustness of up to 3.5 mm long Photonic Needles with cross-sectional dimensions down to 5 μm wide by 10 μm thick. These Photonic Needles could be used, for example, as light delivery probes for multi-photon imaging, excitation and collection, or as a substrate for much smaller waveguides designed for low loss at visible wavelengths. Also the fabrication concepts demonstrated in this work are easily transferrable to numerous other materials and etch chemistries. This platform leverages the mature fields of both micro electro mechanical systems and silicon photonics. In fact, similar needle-like platforms have recently been shown for electrical probing^51–53^, demonstrating how these Photonic Needles could enable a new scalable platform with 100’s or even 1,000’s of light delivery sites and/or other electrical or mechanical elements, by displacing only a negligible volume of tissue.
